# Picture Me Smokefree: A Qualitative Study Using Social Media and Digital Photography to Engage Young Adults in Tobacco Reduction and Cessation

**DOI:** 10.2196/jmir.4061

**Published:** 2015-01-26

**Authors:** Rebecca J Haines-Saah, Mary T Kelly, John L Oliffe, Joan L Bottorff

**Affiliations:** ^1^Institute for Healthy Living and Chronic Disease PreventionFaculty of Health and Social DevelopmentUniversity of British Columbia OkanaganKelowna, BCCanada; ^2^School of NursingUniversity of British ColumbiaVancouver, BCCanada; ^3^Faculty of Health SciencesAustralian Catholic UniversityMelbourneAustralia

**Keywords:** smoking cessation, tobacco use, young adults, Facebook, participatory visual methods

## Abstract

**Background:**

Young adults have high rates of tobacco use compared to other subpopulations, yet there are relatively few tobacco interventions specifically targeted to this group. Picture Me Smokefree is an online tobacco reduction and cessation intervention for young adults that uses digital photography and social networking.

**Objective:**

The main goal of the project was to determine the feasibility of engaging young adults in participating in user-driven, online forums intended to provide peer support and motivate critical reflection about tobacco use and cessation among this high-use, hard-to-reach population. A related aim was to explore the influence of gender-related factors on participation, in order to determine the need for online interventions to be tailored to the specific gender preferences reflecting young men and women’s participation styles.

**Methods:**

A total of 60 young adults ages 19-24 years who self-identified as current cigarette smokers or who had quit within the last year were recruited from across British Columbia, Canada, and participated in an online photo group on Facebook over a period of 12 consecutive weeks. A variety of data collection methods were used including tracking online activity, a brief online follow-up survey, and qualitative interviews with study participants. Data analysis involved descriptive statistics on recruitment, retention, and participation and qualitative (eg, narrative analysis, synthesis of feedback) feedback about participant engagement.

**Results:**

Findings from this study suggest good potential for Facebook as an accessible, low-cost platform for engaging young adults to reflect on the reasons for their tobacco use, the benefits of quitting or reducing, and the best strategies for tobacco reduction. Young adults’ frequent use of mobile phones and other mobile devices to access social networking permitted ease of access and facilitated real-time peer-to-peer support across a diverse group of participants. However, our experience of conducting the study suggests that working with young tobacco users can be accompanied by considerable recruitment, participation, and retention challenges. Our findings also pointed to differences in how young women and men engaged the photo-group intervention that should be considered, bearing in mind that in follow-up interviews participants indicated their preference for a mixed gender and “gender neutral” group format.

**Conclusions:**

Tobacco interventions for youth and young adults should be embedded within the existing social networking platforms they access most frequently, rather than designing a stand-alone online prevention or intervention resource. This subpopulation would likely benefit from tobacco reduction interventions that are gender-sensitive rather than gender-specific.

## Introduction

### Background

In Canada, young adults have the highest prevalence of smoking compared to other age groups. Moreover, the prevalence rate almost doubled from adolescence (11%), with an overall smoking rate of 20% for the 20-24 year olds in 2012, according to the findings of the Canadian Tobacco Use Monitoring Survey. Within these subgroups of tobacco users, gender-related differences in smoking were also notable in that they were consistently and significantly higher among young adult men compared to young adult women (23% and 17% for men and women ages 20-24 years, and 27% and 17% for men and women ages 25-34 years) [1].

While the bulk of tobacco prevention and intervention efforts have been directed toward school-age children and adolescents, less attention has been paid to targeting cessation strategies to young adults [2]. Related to this, young adults have cited the need for relevant, creative interventions that respect their choices and emphasize the positive aspects of quitting [3]. In the absence of targeted efficient cessation interventions, young adults who smoke have remained a subgroup at higher risk for continued smoking [4]. Likewise, while tobacco researchers have prioritized investigations of how gender-related factors influence smoking cessation [5], very little is known about how gender influences tobacco use by young adults. In the current study, we set out to address this knowledge gap by distilling how best to support the reduction and cessation efforts of young adults ages 19-24 years through the trialing of social networking and digital photography as novel tobacco intervention tools with this subgroup. Our aim was to utilize capacity from an existing social network platform to develop a user-driven, online community that supported and promoted cessation and being “smokefree” based on users’ photo posts and dialogue.

### Prior Research and Interventions

Social media and social networking sites (SNS) may provide opportunities to engage young adults in tobacco reduction and cessation through the use of accessible and scalable technologies that enable user-driven participation and interaction. Using the Internet to deliver innovative cessation programming has a longstanding history in Canada where, in 2004, the term Web-Assisted Tobacco Interventions (WATI) was coined to refer to smoking cessation delivered online and through personal mobile and wireless devices. In 2004, the WATI Initiative was formed to support the research, development, and delivery of tobacco cessation interventions across Web-based and mobile platforms [6]. Recognizing the potential to move beyond the delivery of “static” educational materials or didactic online resources, the WATI initiative in tobacco control anticipated what has now become standard in the delivery of eHealth programming, as it emphasized the need for interactive, user-driven technologies in order to engage people in health promotion interventions. Yet in a review of WATI, it was suggested that tobacco intervention websites had underutilized online capacity in terms of harnessing the potential for interaction and for personalizing cessation and reduction treatments for users [7].

The United States has provided additional, prominent examples where online presence and social media were used to engage youth on tobacco issues, such as the American Legacy Foundation’s longstanding “The Truth” [8] initiative, which mobilized young people in advocacy against tobacco corporations, and the more recent “Smokefree Teen” [9] cross-platform, online initiative for adolescents from the US National Institutes of Health and the National Cancer Institute. In both the United States and Canada, tobacco control and health promotion organizations have regularly sponsored online contests where youth create their own anti-smoking visual media, by making and sharing photos or videos, such as the “Let’s Talk Tobacco” [10] project from the Canadian Cancer Society. In British Columbia, Canada, the context for the current study, the province’s young adult tobacco prevention initiative, Quitters Unite [11] (funded by Health Canada and the Heart and Stroke Foundation), successfully ran a photo contest in 2011 based on the theme “My Smoke-free Lifestyle”, [12] where over 150 entries were received for a CAN $300 cash prize, the approximate cost of buying cigarettes for 1 month. Specific to young adults and SNS capacity, in 2014 the Canadian Cancer Society (in partnership with Health Canada) launched a website, a cross-platform social media campaign, and mobile app under the banner “Break It Off” [13] to encourage young women and men to “break off their relationship with smoking and stay smoke-free” [14]. Specific to SNS, a unique feature of Break it Off was an add-on app for use within Facebook, which participants could use to change their “relationship status” and announce to their Facebook network that they had ended their relationship with smoking (similar to the feature where Facebook users indicate a change in their romantic relationships by linking or de-linking to another user), as a way to lobby social support for sustaining their quit [15]. Also targeting young adults, the Crush the Crave mobile phone app for young adult cessation [16] (a partnership between tobacco researchers at the Propel Centre for Population Health Impact at the University of Waterloo in Ontario and the post-secondary anti-tobacco initiative “Leave the Pack Behind” from Brock University, Ontario) has been aimed at providing young adults with tools to track and log their cessation attempts, in addition to ways to share their successes and receive support and reinforcement from friends and family via SNS. An analysis of the content posted to the Facebook page promoting the Crush the Crave app has suggested that people who commented on posts (ie, replies) were highly engaged with the visual content and images posted (posts were made almost entirely by page moderators) and that the majority of comments (77%) were themed around showing support for cessation [17].

Consumer research on social media use in the province where this research project was carried out has suggested that among British Columbians ages 18-29 years, 76% have used Facebook on a daily basis [18]. Facebook’s own statistics on usage by Canadians suggest that they are the most active users of the platform globally, with over 14 million users logging into their accounts daily and 9.4 million of those doing so from mobile devices [19]. There is also evidence to suggest differences in how women and men engage online and that gender norms shape how they participate in SNS. For example, women have been shown to be more active users of social media for staying connected with others and for “relationship maintenance” when compared to men [20-22]. Relevant to our project, age and gender differences are also seen in SNS use where “women and the young drive Facebook usage” [23]. Studies with Facebook users have shown that women posted more photos of themselves on Facebook as well as updated their status, commented on posts, and endorsed posts by others more often than did men [24,25]. These differences in use are seen on other SNS platforms; for example, women also far outnumber men as users on the image-based platform Pinterest (72%), whereas men outnumber women on YouTube, with 68 million more monthly visits [25-27].

While there is sizable research on delivering online and mobile phone cessation supports for adult populations, review studies have shown inconsistent evidence for effectiveness [28,29]. Similarly, studies have suggested that text messaging [30,31] and peer-based email support [32] for cessation have had good potential for engaging young adults and have been effective for reducing tobacco consumption [33,34] and for smoking cessation in the short term [35]. In their systematic review of WATI for young adult health behavior change, Crutzen et al [36] considered 26 studies of Web-based interventions for adolescents or young adults ages 12-25 years, with 8 of 26 studies that were specific to smoking cessation. An important conclusion of this review for Picture Me Smokefree was that strategies for generating online peer-based support such as discussion boards were only moderately used, yet overall it is still difficult to ascertain the effectiveness of various methods used to increase exposure to online interventions because tracking and reporting exposure measures was inconsistent or non-existent [36].

We are unaware of any studies where the primary delivery of a WATI is through an existing SNS platform such as Facebook, especially where the content is generated by participants (ie, rather than delivered “to” them by researchers on “intentionally created” websites or intervention communities) [37]. Despite the belief that delivering tobacco interventions through SNS may be well suited to a demographic where using sites such as Facebook has been woven into daily life for many, to date, there has been little research specific to young adults and the delivery of SNS-based cessation interventions [38]. A systematic review of randomized controlled trials (RCTs) of WATI from 2011 concluded that Web-based interventions had the potential to offer low-cost, wide-reaching treatment [39]. Yet Hutton et al [39] identified only one RCT carried out with young adults [40], which had multiple components that made it difficult to evaluate in terms of isolating the efficacy of the Internet for tobacco cessation. The RealU online intervention for college students sent weekly emails to participants inviting them to the intervention website where they were asked to report on their health behaviors of the previous week, take an interactive quiz related to smoking dependence, and read online general interest articles [40]. The authors reported higher 30-day abstinence rates in the control group, but not continuous 6-month abstinence rates. Another feasibility study with 46 college students in the United States evaluated a mobile phone text-messaging smoking cessation program [41]. The text messages were individually tailored, based on personal smoking information that the participants provided by answering assessment questions on a website. Participants recorded their daily smoking behaviors on a website and could access quitting information there. Although 22% quit smoking after 6 weeks in the study, the attrition rate was 33%. The authors replicated this study in 2008 with 31 college smokers who wanted to quit, concluding that phone text messaging held good potential as a tobacco intervention with young people [42]. A larger RCT with 1705 smokers (mean age 25 years old) also tested the effectiveness of mobile phone text messaging as a cessation tool [43], reporting that 28% of the intervention group had quit at the 6-week mark. Indeed, at 26 weeks there was little difference between the intervention and control groups, because the control group began reporting increasing quits. In all these studies, the intervention was delivered without communication or support being generated among youth or young adult participants, and smoking abstinence, as distinct from tobacco reduction, was the measured cessation outcome.

### Research Questions and Feasibility Indicators

To determine the feasibility of using digital photography and SNS as a smoking cessation and reduction intervention for young adults, the current study was guided by three core elements of the framework developed by Bowen et al [44], summarized in Table 1.

Given the presence of gender-related influences in smoking prevalence for this age-based subgroup, we also collected findings disaggregated by gender, to provide a preliminary assessment of the extent that gender-related factors may have influenced participants’ use of our online and photo-based intervention.

**Table 1:** Study feasibility foci and outcomes of interest (adapted from Bowen [44]).

General areas of focus	Specific areas of focus	Outcomes of interest
Acceptability: To what extent was the intervention suitable for attracting the target audience?	Clarity and suitability of the study recruitment strategies and eligibility criteria	Number of inquiries vs number of sign-ups
		Number of inquiries outside the eligibility criteria
		Findings disaggregated by gender
Implementation and practicality: To what extent could the intervention be implemented given possible resource constraints?	Ease and cost of recruitment and participation	Count of participants recruited weekly
		Recruitment sources and cost analysis
		Remuneration costs
		Findings disaggregated by gender
Demand: To what extent were aspects of the intervention actually used by participants?	Participant engagement	Level of photo group activity
		Completion and retention rates at 12 weeks
		Survey responses and rates
		Participant interviews
		Findings disaggregated by gender

## Methods

### Study Design

A prospective, non-comparative design was used. This design is appropriate for feasibility studies that aim to determine whether the intervention is appropriate for further testing and estimate important parameters that are needed to refine the design of a full scale study [44,45]. The study protocol was approved by the ethics review board at the University of British Columbia, Vancouver, Canada.

### Picture Me Smokefree Intervention—Rationale and Implementation

To facilitate user-driven peer support and interaction within the context of an online group, the design of our project adapted Photovoice [46,47], a visual method that is well established in health research, tailoring it for use with digital photography and an SNS platform. The broad intent of using what we have termed “participant-driven” photography methods in public health research has been to mobilize participants’ creativity and community engagement beyond what is possible through using narrative-based methods (ie, interviews and focus groups). Conventionally, participant-driven photography projects have been community-based in neighborhoods or other physical settings for health promotion, aiming to collectively empower research participants using photography to document local health issues. In “true” Photovoice research, there is an active community advocacy component where participants work together and make collective decisions about the photo-based findings and themes they want to share with stakeholders and other decision makers at the project’s conclusion. Participant-driven photography has also been used to engage participants in critical reflection about health behaviors and illness experiences at an individual level, through integrating discussions about photographs within interviews conducted between participants and researchers [48,49].

**Figure 1:**
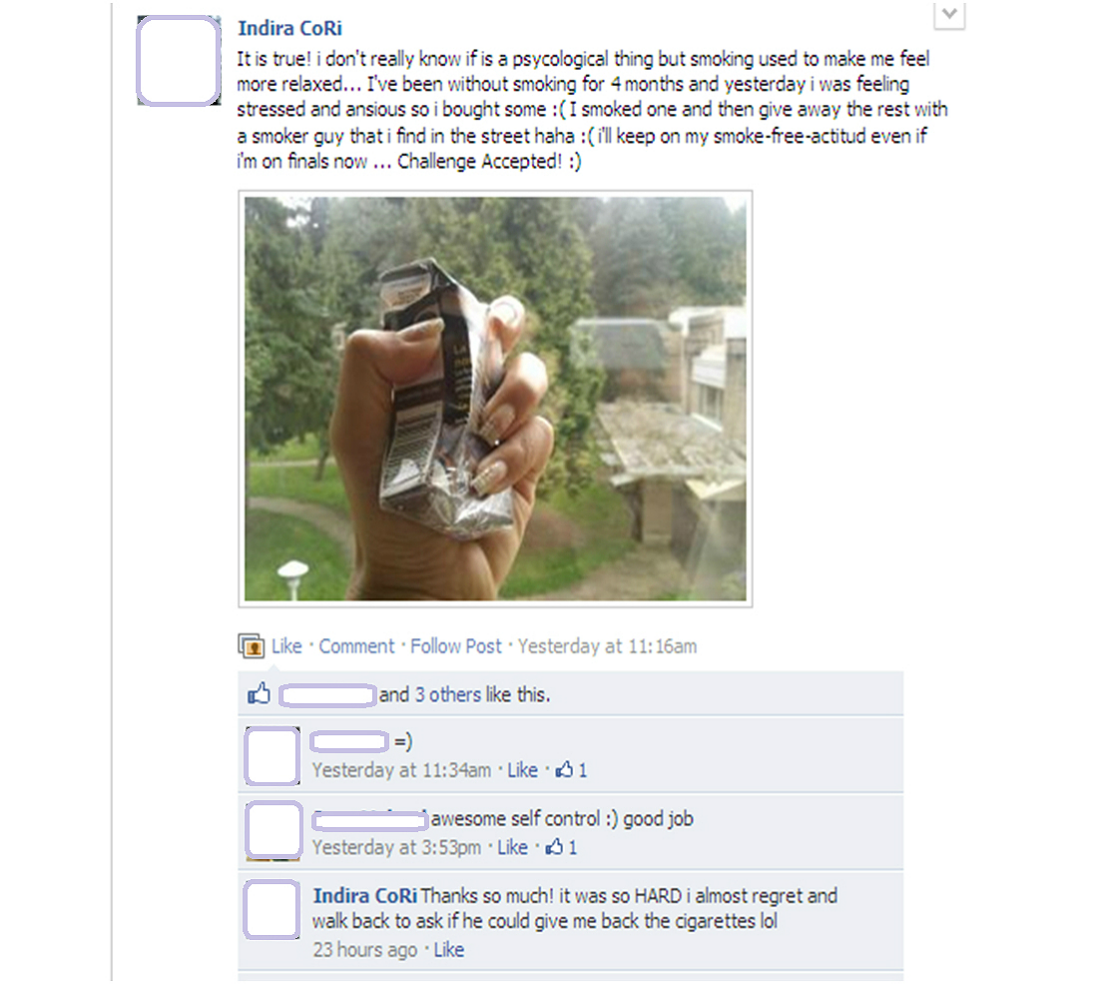
Screenshot from the Picture Me Smokefree photo group on Facebook.

The investigative team for this project had previously conducted several participant-driven photography studies to understand the experiences of people that smoke, focusing specifically on how gender-related factors influenced tobacco use and the need to prioritize gender considerations in cessation and reduction interventions [50-52]. Departing from the usual approach in participant-driven photography research of providing participants with disposable cameras, Picture Me Smokefree was designed to assess the potential for adapting photo methods to a social networking platform, capitalizing on young adults’ familiarity with documenting and sharing daily activities through digital photography (camera and mobile phone). A key difference in the research process for Picture Me Smokefree compared to our past participant-driven photography studies was that by asking young adults to take pictures using their own digital cameras or mobile devices and to caption them online, the balance of power shifted to the participants and the researchers’ involvement in assisting them with the process of taking and sharing photographs was minimal. As a result, interactions between project participants were instantaneous and for the most part unfiltered between members of the online photo group created for the project, and the researcher participated as a group member rather than moderator (Figure 1). Most topics were user-initiated, but occasionally the researcher-moderator posted photo challenges, topics, or “mini-contests” to engage participation during periods of low activity within the photo group.

Instead of creating a standalone website specific to our photo-based intervention, we chose to host our project on Facebook as a low-cost option with the potential to engage young adults who have been the most frequent users of this SNS. The built-in features of Facebook allow users to post and comment on photos in a variety of spaces (ie, pages, groups, and walls), creating spaces for dialogue, debate, and online support. While we initially planned for the Picture Me Smokefree photo-posting group to be open for viewing by the public, once recruitment was underway we elected to create a private “members only” group on Facebook. This decision was based on users’ stated preferences for sharing their photos about smoking and cessation in a limited capacity, so that posts and other content they shared could not be seen by their network of Facebook contacts or be viewed publically (ie, especially family or employers who may not be aware of their smoking status).

### Recruitment

**Figure 2:**
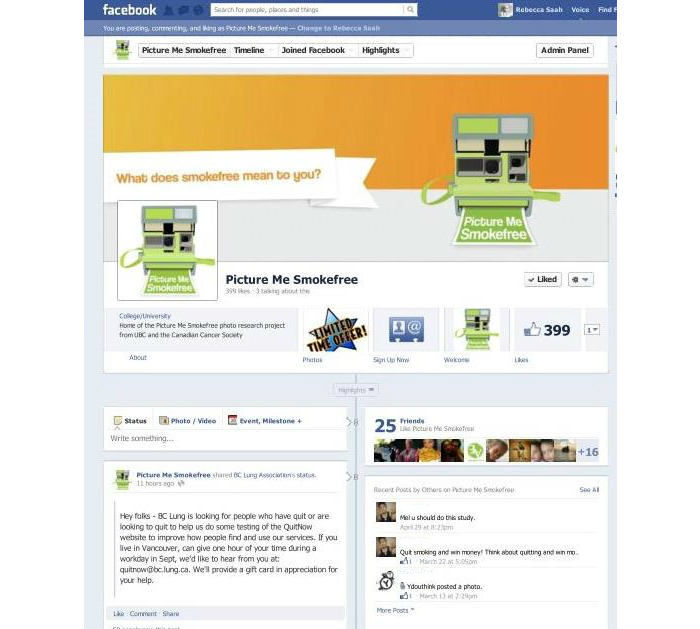
Picture Me Smokefree Facebook landing page.

**Figure 3:**
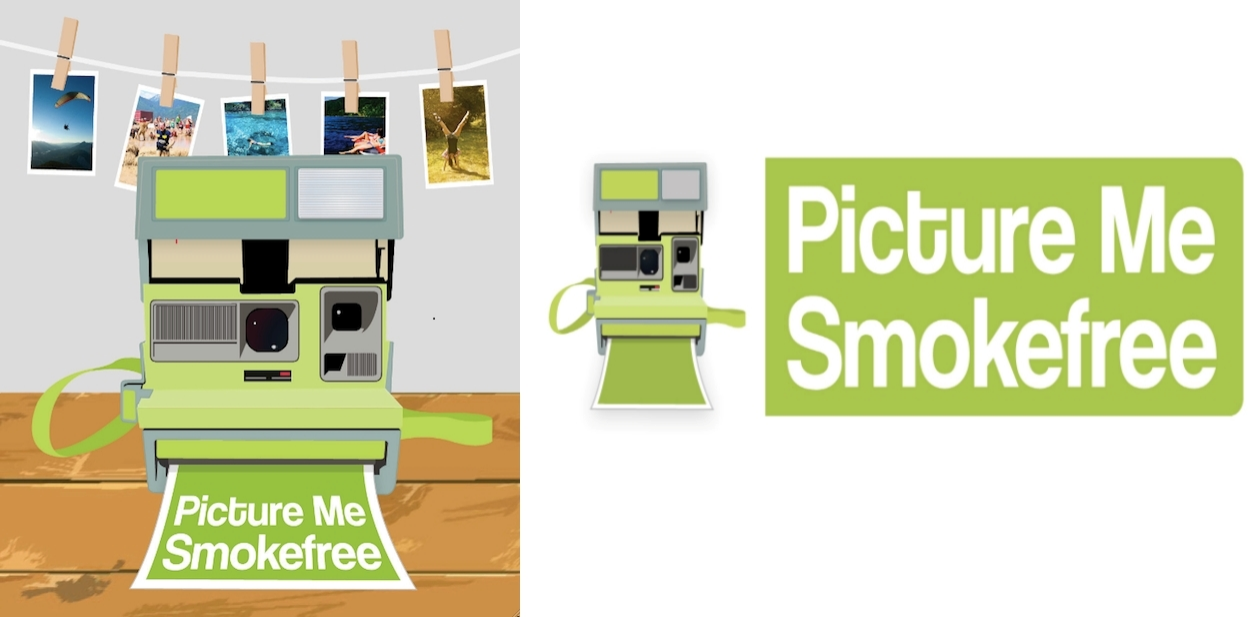
Picture Me Smokefree intervention logo and social media branding.

The target for recruitment was 60 young adults. The eligibility criteria included being a man or woman aged 19-24 years, living in British Columbia, having English literacy, and having Internet and camera access. Although we had resources to provide participants with cameras, only 3 people required this. Recruitment was open to active smokers (with some interest in quitting) or those who had quit within the last 12 months. We used a variety of recruitment strategies beyond social media, although a recurring Facebook ad garnered the majority of inquiries. Separate from the photo-group space, we created a Facebook “landing page” (Figure 2) for the study, which provided general information and a way to promote recruitment. In addition to the standard Facebook features of the public wall or timeline where posts could be shared, our Facebook page included an online Web form where participants could sign up for the study (responses were pushed to us via email). Additionally, we contracted the services of a youth-friendly social marketing firm to develop a consistent brand identity (ie, logo, color scheme) for the project and to promote the study through online and offline channels (Figure 3).

We also worked with a team of community-based health promoters to distribute flyers, posters, and magnets advertising the study to young adults in their local communities across the province. A Twitter page for the study was created to promote the study and to interact with other local tobacco control and health promotion initiatives geared toward youth and young adults. Other recruitment activities included flyers posted on post-secondary campuses and at youth-serving health and social service agencies, print ads in campus newspapers, participants’ referrals, posts on craigslist, and email group “blast” messages sent through professional mailing lists.

### Data Collection and Analysis

To be participants, individuals had to join the Facebook photo group, which first involved brief contact with the study investigator by telephone or in person to provide their informed consent and to receive a brief orientation to the project. Because we know that smoking status may fluctuate often for young adult tobacco users, participants were asked to post to the group for 12 weeks and to make a minimum of one posting per week, preferably by posting an original photo and caption about their experience of smoking or quitting. We defined a participant as active as soon as they had completed one action in the Facebook photo group, posting a photo or comment, sharing a link, or “liking” other posts (Facebook’s “thumbs up” endorsement action). For each week that a participant was active, they received a CAN $10 credit, which was paid out to them at the end of the 12-week consecutive period. Research staff enrolled participants and monitored their activity in the photo group on a weekly basis, manually tabulating the number of posts to the group made by each person. Weekly reminders to post and updates on their current status (ie, credits and weeks remaining) were sent to participants via Facebook and by email. As recruitment was ongoing and participation was staggered over the course of the 10 months that the study was running, the numbers of people within the photo group varied at any given point. The largest number of members in the group was 46, keeping in mind that some people signed up but failed to join the group, and others joined but never posted (study dropouts).

Other data collection measures included a smoking history and demographic form completed at sign-up. At the end of the 12-week participation period, participants were also asked to complete a brief (10 question), anonymous, online evaluation survey, and invited to provide additional feedback about their experience of participating in Picture Me Smokefree in a feasibility interview (face-to-face or by phone). The interview was optional but was offered to all participants who were active in the photo group for at least 1 week and was completed by 22 people (12 men, 10 women). Data analysis involved descriptive statistics on recruitment, retention, and participation and qualitative (eg, narrative analysis, synthesis of feedback) feedback about participant engagement. Multimedia Appendix 1 (slideshow) provides further detail about the recruitment materials, study procedures, and the study results.

## Results

### Acceptability

There were 134 inquiries about participation in the study received: 78 (58.2%) from men and 53 (39.6%) from women. Three inquiries (2.2%) were received by email, and the sender’s gender was not specified. Only 20 (14.9%) of inquiries were outside of the advertised eligibility criteria, suggesting that the recruitment materials and strategies were clear and appropriately targeted to our population of interest. For those instances where the person who inquired was deemed ineligible to participate, the most frequent reason was being outside the age range (n=13), with 1% under age 19, and 11% older than 24. As participants ranging in age from 31-70 years inquired about signing up, this possibly suggests that a photo group on Facebook may also be attractive to adults who smoke or who want to quit. Only a few people that contacted us about the study (n=6) booked an appointment to sign up but did not follow through. For other inquiries lost to follow-up, we assumed general disinterest. Only one person who contacted us explicitly declined to sign up because they did not have a Facebook account.

Through the recruitment process, an unanticipated issue arose regarding confidentiality and privacy. Although the study was targeted directly to young adults, we received a few inquiries from parents, relatives, and service providers seeking to sign up on behalf of a young adult who smoked (n=5). This speaks to the need for greater clarity in our recruitment materials regarding participation as voluntary and direct and to have explicit guidelines in place for research staff when third-party inquiries are received. Another issue was that one-quarter of our participants (8 women, 7 men) opted to create an anonymous profile or “Facebook alias” user account—an option that we encouraged as part of our informed consent process—due to privacy concerns and to ensure that their identities would not be associated with a study about tobacco use.

### Implementation and Practicality

We enrolled 60 individuals aged 19-24 years (mean age 21 years) into the study over a period of 10 months in late 2011, through until the summer months of 2012. With 34 men (57%) and 26 women (43%) signing up for the study, the gender split reflected patterns seen for the study inquiries. Recruitment occurred more slowly than we anticipated, which posed a significant challenge for meeting the pre-determined feasibility targets for signing up 5 new participants per week. Since we attracted very few participants (≤3) in the first months of the study, we refined and expanded our initially proposed strategies for reaching young adult tobacco users. To this end, the first few months constituted a “soft launch” on Facebook, while we determined the best combination of online and offline approaches to use. Recruitment ceased in July 2012 when we reached our sample target (N=60). There were only 3 months when there were more than 5 new participants that signed up: March (19), February (11), and June (10). While these increases in recruitment were arguably tied to intensification and diversification of our efforts, we hypothesized that they may also be tied to events in the local post-secondary academic calendar (ie, vacation, mid-term examinations), as many of our participants were students.

Overall, about half of the total inquiries (68/134, 50.7%) received about the study came through targeted Facebook ads, which we set to be shown to all Facebook users that met our age and geographic inclusion criteria; to a lesser extent targets to users with profiles associated with post-secondary institutions and terms referencing tobacco use or cessation were also used. After Facebook, the top three *referral sources* (ie, how potential participants heard about the study) were from a friend participating in the study (21/134, 15.7%), from a mass email message they received about the study (9/134, 6.7%), or from a flyer posted at a youth-serving agency (9/134, 6.7%). Considering only those inquiries that progressed to sign up as participants, recruitment from Facebook (17/60, 28%), friend referral (17/60, 28%), and posted flyers (16/60, 27%) accounted for most (50/60, 83%) of our final sample. Male participants were more likely than female to be referred to the study by a friend who had signed up to participate (11/34, 32% vs 6/26, 23%). It is also noteworthy that there were 10 participants (5 couples) who signed up and had a partner also participating, which may speak to the importance of considering intimate partners as a source of support for young adult smoking cessation, in light of studies suggesting that gender relations are key influences on cessation for adult heterosexual couples and families [53-55].

In terms of the *referral type* (ie, the method used for contacting us), most participants used the online contact form on our Facebook landing page (25/60, 42%), sent a direct email (13/60, 22%), or telephoned (10/60, 18%). For those who signed up for Picture Me Smokefree, there were some gender differences noted in the contact methods used, however, as men where 3.5 times more likely to communicate initially by text messaging, and women were twice as likely to use email.

The major costs associated with recruitment were graphic design services, printed materials, newspaper advertising, Facebook ads, and contracted social marketing and community recruitment services. At approximately 15% of the total budget, recruitment costs were reasonable, although perhaps higher than typical given the relatively small number of participants recruited. Yet given that our population of tobacco users is seen as a “harder-to-reach” group, we believe the expense was justified, while recognizing that as recruitment occurred as a process of “trial and error”, these costs could most certainly be streamlined in the context of future studies.

### Demand

To ascertain demand and participant engagement, it is useful to consider the characteristics of those who signed up (smoking status, gender), how they participated in the group (type, frequency of their activities), and 12-week study completion rates (attrition). Finally, we considered the findings from an anonymous survey at the conclusion of the participation period, in order to summarize user feedback on the experience of being a participant in Picture Me Smokefree.

Picture Me Smokefree attracted individuals with a wide range of self-reported smoking patterns at the point of signing up: from light to heavy daily smokers, weekly and occasional social smokers, as well as a few who were attempting to maintain their quit. Almost half (25/60, 42%) of the participants reported being daily, light smokers and consuming 5 cigarettes or less per day. Those who were classified as heavier smokers, at between 15-20 cigarettes per day, comprised 15% (9/60) of participants. Like the range in their daily smoking frequency, the length of time participants had smoked varied widely; the most common length of time participants reported having smoked was from 3-5 years. Additionally, Picture Me Smokefree participants reported that their smoking status changed over the course of the 12 weeks they participated. For example, among the participants who completed the online follow-up survey (39/60), 10% (4/39) reported they had quit during their participation and 51% (20/36) had reduced their smoking during the course of the study. For another 24% (9/36), there were no reported changes, and 5% each (2/39) stated they had relapsed, increased tobacco use, or had already quit prior to the study. Overall smoking status among the women and men recruited was similar: 27% (7/26) of women and 29% (10/34) of men characterized themselves as actively quitting, and 73% (19/26) of women and 71% (24/34) of men were smokers at their sign-up. As the final survey was anonymous, we did not collect findings about gender in regards to changes in smoking status over the 12 weeks.

Although Picture Me Smokefree attracted more men than women to participate, overall women participated more frequently and posted more content to the photo group than did men. We tracked the level of study participation and learned that women were more likely than men to complete more weeks of the study and post to the group at least once during each of the 12 weeks. Table 2 shows the differences in the level of participation for men and women, with reported percentages weighted by gender. While women were more likely than men to be high participators, more men than women were seen in the medium participator group. However, collapsing the medium/high distinction, any differences all but disappear, with 70% (24/34) of the men and 69% (18/26) of the women participating in the Picture Me Smokefree photo group for between 5-12 weeks. Low participation in the study and dropping out of the study was not very different for men and women. In terms of attrition, we classified one-fifth of participants as study dropouts (19% of women and 21% of men) as they either failed to join the Facebook group after signing up with the study investigator, or joined the Facebook page and posted only once or not at all. Although the retention rate was better than our pre-set target of 75% and suggests that engagement in the photo group was strong, a greater number of both men and women in the high participation group would have been more desirable.

**Table 2:** Study participation of men (n=34) and women (n=26).

Participation		Men, n (%)	Women, n (%)
Percentage of Picture Me Smokefree participants		34 (57)	26 (43)
**Participation rates**			
	High participation (9-12 weeks)	15 (44)	17 (65)
	Medium participation (5-8 weeks)	9 (26)	1 (4)
	Low participation (1-4 weeks)	3 (9)	3 (12)
	Study dropouts	7 (21)	5 (19)
Total		100	100

**Figure 4:**
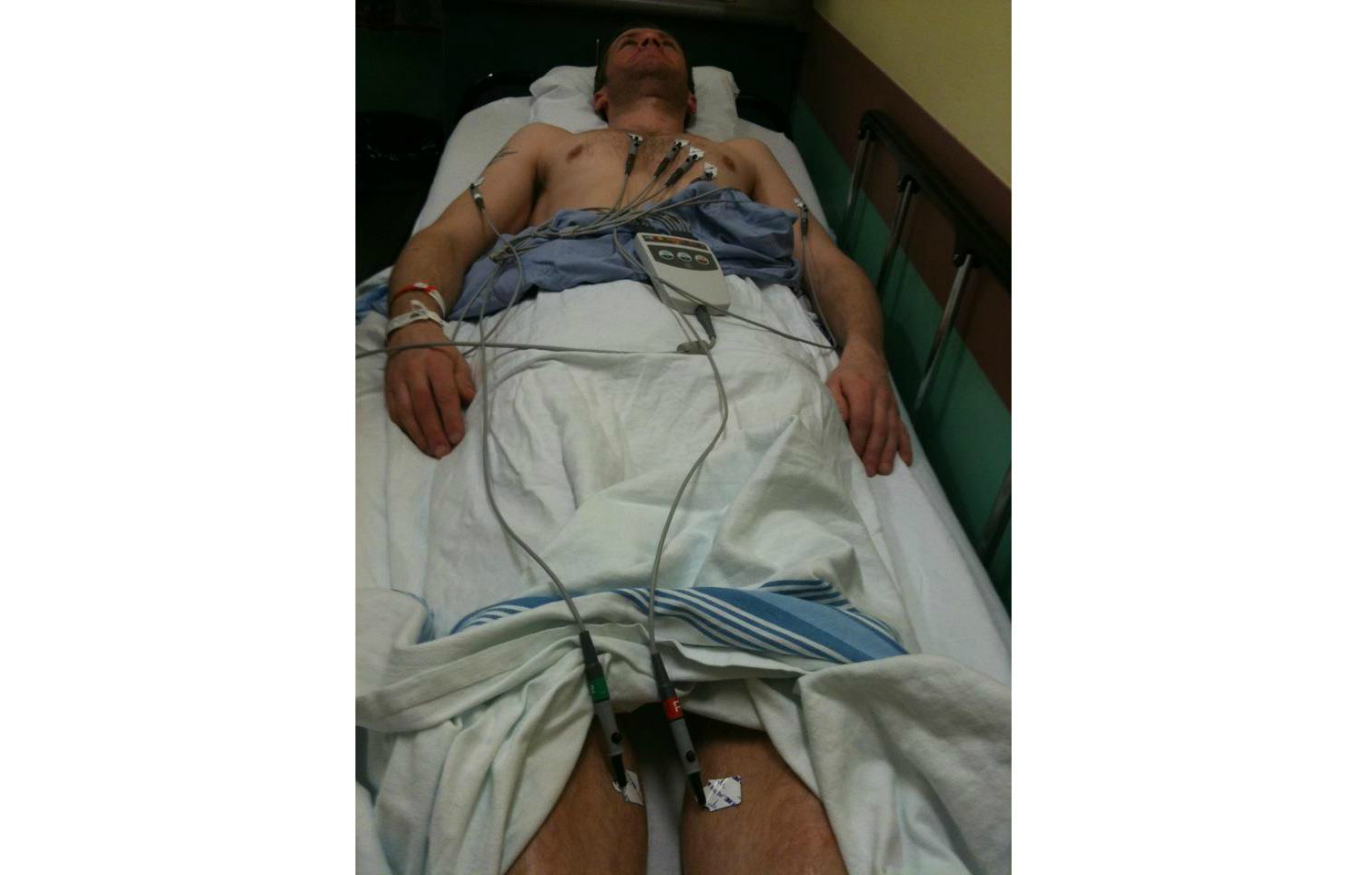
Example of a participant photo and caption about tobacco use, health, and quitting (Woman): “This is why I hate smoking. They said his heart is inflamed and smoking is contributing to it. I know that if we kept smoking this is where we will end up. I just keep asking why would I do this to myself?”.

**Figure 5:**
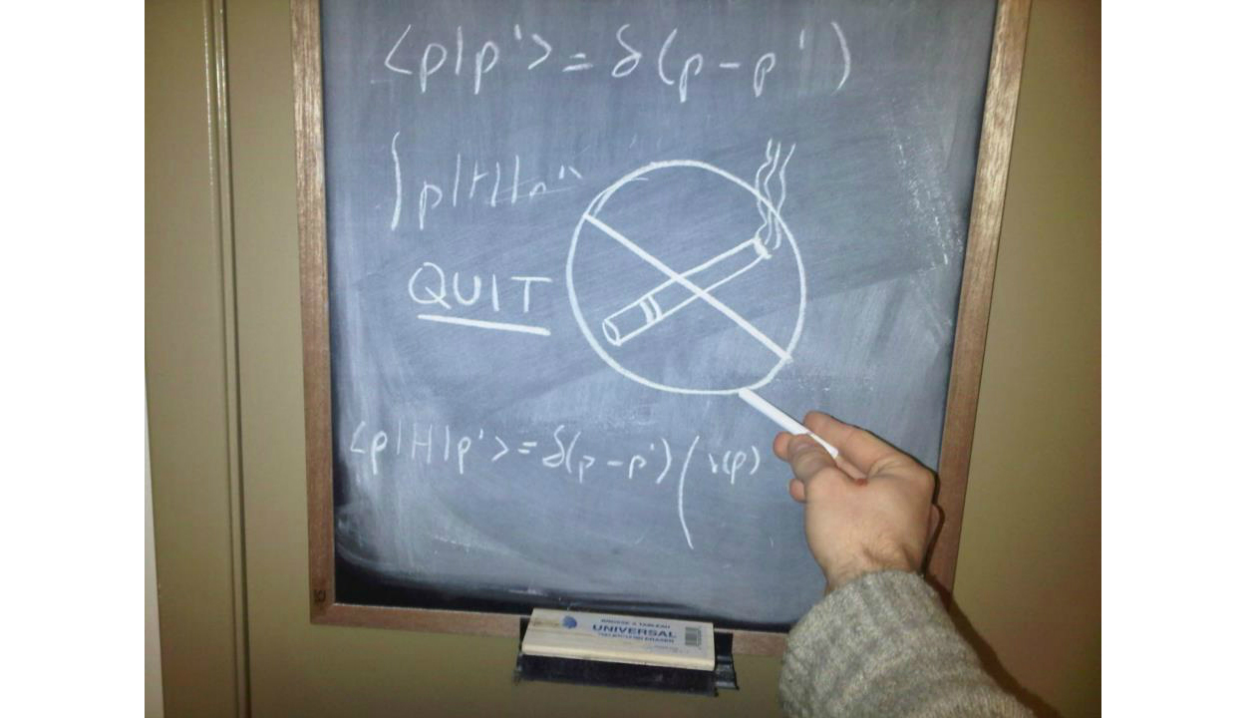
Example of a photo and caption about tobacco use, health, and quitting (Man): “Sometimes smoking gets in the way of my academic pursuits. I take too many breaks, I put off time for studying by adventuring to the local mini mart of gas station, I relax with the subtly deadly nature of tobacco. I feel that if I don’t quit soon, it’ll harm me in the long run, and my pursuit of becoming a professor will die off quit quickly (and literally). As soon as exams are out of the way, I will return to my non-smoking ways. But until then, I must cope”.

Posts to the photo group were tracked both manually by a research assistant and electronically through NVivo NCapture software, which was used to export Facebook data at the completion of the study. In total, there were over 1800 actions (including photos, comments, “Likes”, and “shares”) to the photo group by participants, which indicates reasonable engagement for a group of this size. Picture Me Smokefree participants posted 283 photos, with 94 photos posted by men and 189 by women. This difference holds when looking at the number of postings by individuals, with a mean of 9.45 photos for women and 4.27 photos for men. Of the 18 participants who did not post any photos (but commented and endorsed other content in the group), 6 were women and 12 were men, indicating the same trend: overall women were more likely to post photographs. While we saw rates of activity that were comparatively higher for women than for men, quantity of interactions should not alone be the markers of significant engagement. For instance, although some men posted less frequently, this did not appear to be related to a lack of interest in sharing or “connecting” online. Both women and men posted photos and captions about their experiences with tobacco use and cessation that were reflective and deeply personal, often sharing details about tobacco use and their struggles with quitting in the context of their family life and relationships, and as linked to work and school settings. Two examples illustrating this point are provided by Figures 4 and 5.

**Figure 6:**
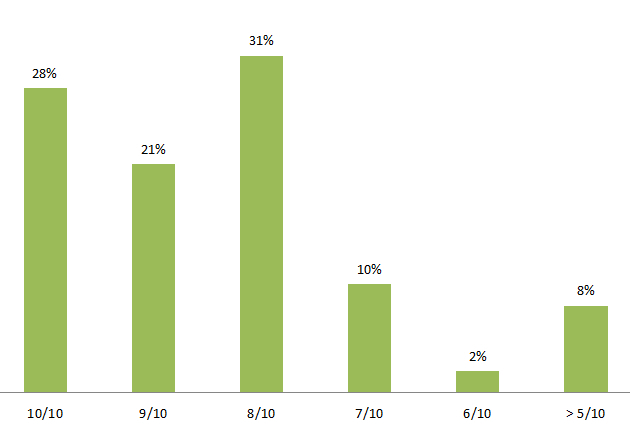
Follow-up survey question: “On a scale of 1-10, please rate your overall experience as a participant in this project”.

The response rate for the brief, online follow-up survey at study completion was adequate at 65% (39/60). When asked to rate their “overall experience” on a scale of 1 to 10, many participants (31/39 or 79%) provided a rating of 8/10 or higher (Figure 6). Similarly, when asked “would you participate in a project like this again?”, all but 1 respondent predicted they would (38/39). Completion of the survey also appeared to be tied to higher levels of participation and engagement in the study, as most of those who completed the survey (32/39) indicated that they had participated between 9-12 weeks. Respondent recall for this number is likely accurate, given that all participants were provided with a report on the number of weeks they were active in the study, as this was linked to their weekly credits and the amount of their final honorarium payment.

To briefly summarize narrative (qualitative) feedback from the online survey and the one-to-one interviews (22/60), most men and women indicated that what they viewed was positive, non-judgmental, and endorsed the supportive tone of the group, especially as it was presented in the context of interactions between moderator and participants, between group members who had quit and those who were still actively smoking, and also as it was expressed toward those who had relapsed (see Textbox 1). Yet a small number of those who had quit or were planning to do so expressed that they would have preferred an “all quitters” group because they found that some of the posts by active smokers were “too pro-smoking” and shifted the tone away from reduction and cessation.

**Themes in participants’ feedback (survey and interviews).**

Picture Me Smokefree participants liked:

- support and connections with peers
- new information and diverse views represented
- freedom/creativity in posting
- honesty/non-judgment
- private group (smoking status not visible to public)
- easy: “only a mild hassle”, “easy money”
- reinforcement for cessation (alerts creates daily reminders)

Picture Me Smokefree participants’ suggestions for improvement:

- more interaction between members in the group
- more interactive and structured activity (eg, surveys, contests)
- alerts or reminders to post
- too busy/lost interest (increase engagement)
- dislike Facebook (provide other online options)
- add offline support meetings
- help with ideas for creating photo posts

In regards to how Picture Me Smokefree might have positively influenced their movement toward tobacco reduction or cessation, participants provided the following qualitative feedback:

Um, I thought it was a really great experience, it really opened my eyes to what other people think and associate with smoking…And I think it was great to see what everybody else’s ideas and their pictures and stuff and it really made me personally think of why I smoke and what the reasons are and what else I associate with smoking as well.

woman, age 23

I liked having a place where a lot of people might come and put why they still smoke and the triggers that make it harder for them to quit and just kind of bringing more of a general knowledge of the struggle people have when they try to quit smoking.

woman, age 24

I think the best part of about it was the fact that there was a group of smokers or ex-smokers that kind of worked together to help bend or quit the habit and uh it kind of served as a motivation…There was a mini-community thing going on and I really liked that. I liked seeing others and their photos and how they’re dealing with it or what problems they have associated with smoking which, which kind of gives you broader understanding of why people smoke which is really interesting.

man, age 19

Even after the study had ended, participants’ provided feedback (sometimes months later) that Picture Me Smokefree helped motivate them to quit: “I have quit Facebook for the time being. I have also quit smoking” (woman, age 23), “Haven’t smoked since my twelve weeks wrapped up. Instead, I’ve been eating way healthier, working out, and getting a ton more work done towards my career. Off to a great (re)start” (man, age 21), and “I am going to attempt what I believe to by (sic) my first serious attempt to quit smoking. I think the seed of quitting was planted in my mind this year when I took part in your project” (man, age 21).

A further key issue associated with assessing the feasibility of participation in our intervention pertains to practicality and the costs of remunerating participants. For this feasibility study, our costs were relatively low in terms of equipment purchases and payments made to participants. With only three cameras purchased for those without access, under CAN $500 was spent on equipment. It is also notable that Internet access was not a barrier to participation—even for the participants that were marginally housed and recruited from youth shelters and transitional housing, Internet and computer access was readily available to them at these agencies. The total amount spent to remunerate participants was just under CAN $5000 over the course of 10 months, and keeping in mind that not everyone participated on a weekly basis, on average these costs were about CAN $109 per person. There were also 6 participants we repeatedly contacted that did not collect their final payments, for amounts ranging from CAN $10 to $110.

## Discussion

### Principal Findings

The current study findings support the feasibility of adapting photo research methods to an SNS to form a user-driven, online intervention that can provide an opportunity for young adults to reflect critically about their tobacco use and access peer support for cessation. Picture Me Smokefree demonstrated that embedding a tobacco intervention within social media platforms has potential to engage young adults in cessation or reduction plans by drawing on their familiarity and experiences in using Facebook. The strength of this approach appears to be the interactive quality and peer contact provided by social media that is not only engaging but offers young adults the opportunity to learn from each other’s experiences and to provide mutual support, regardless of their smoking or quitting status. However, even when an intervention is hosted in an online context where young adults are frequent and fluent users, we found that recruiting current and former tobacco users from this subgroup can be difficult and time-consuming. A key conclusion of our study is that recruitment efforts need to be creative and multi-pronged (taking place both online and offline), and that this type of tobacco cessation intervention may require considerable resources to attract and retain participants. In this case, reaching our (relatively small) target of 60 participants to an online group required 10 months of experimenting with various recruitment efforts, but we anticipate that in a future study the lessons learned from this preliminary research would likely reduce the time period to attract sufficient numbers considerably. However, any assessment of feasibility must seriously consider that this particular subgroup of smokers might be considered “hard to reach” from an age and cessation-based perspective, and therefore perhaps less likely to join an intervention, regardless of the format.

The design of this study does not permit us to claim generalizability about changes in smoking outcomes associated with our intervention. Nevertheless, the qualitative and self-report findings on how smoking status changed during participation—in particular that over half of the participants reported reducing their tobacco use—suggest that a user-driven photo-group intervention like Picture Me Smokefree shows promise and warrants further study to determine its effectiveness in supporting cessation and tobacco reduction.

### Suggestions for Future Studies

It is clear that some young adults found the online group useful for reducing or quitting smoking, while others may have participated without influence on their smoking status. While most found the context and the content of a group open to participants from a range of smoking statuses to be positive and supportive, there were at least a few participants who expressed a desire for a “quitters only” group, so that they would be exposed only to interactions and content that reinforced their decision to be smokefree. To this end, future comparative studies might compare outcomes when participants are assigned to an “active smoker”, “active quitter”, and to mixed status groups. Although a more detailed analysis of the visual and narrative themes within the photos is beyond the scope of this initial feasibility report, the findings have provided valuable background to inform additional group content and activities (ie, assigning weekly topics or themes for photo posts) that might assist in sustaining engagement in the photo groups and for increasing young adults’ motivation toward tobacco reduction and cessation.

As our study was structured to include weekly credits for participation, it would be particularly important for future research to test alternate mechanisms for incentivizing participation and retention. For instance, a comparison of the weekly payment credit system used by Picture Me Smokefree with that of a photo contest format or another more cost-efficient mechanism of remuneration would be useful. Where regular incentive strategies are generally seen as cost prohibitive when rolling out an online intervention at the population level [36], a virtual reward or contest system (eg, one contest entry per week of activity) would likely be the most appropriate and cost-effective strategy to encourage participation. Still, based on our findings that several participants did not collect their final payments, it is perhaps also the case that monetary incentives were not the prime motivation for participation by all young adults in this study.

This study also provided some preliminary findings to suggest support for interventions that are sensitive to or tailored to gender-related influences, as indicated by some of the variations observed in the online participation by young men and young women in Picture Me Smokefree. Although men and women may have used the group in different ways and with different levels of participation, the follow-up surveys and interviews with participants indicated that both men and women experienced Picture Me Smokefree as helpful because it provided a supportive, non-judgmental online space where they could share quitting and smoking experiences and struggles. The men and women used the online group and the process of posting photos to reflect on their smoking, their habits related to continued smoking, and the benefits of and reasons for quitting. Rather than suggesting support for segregating users into gender-specific cessation or reduction groups, this population may benefit more from reflecting on their own smoking behaviors and from supporting others in a mixed-gender setting.

In an effort to be credible and appropriate for youth and young adults involved with tobacco, there are several other popular image-based social media platforms such as Instagram or Tumblr where an eHealth intervention component might be embedded. Based on feedback from our participants, future revisions for a larger study should include more interactive features in an online group (eg, a group chat feature, weekly group photo topics or assignments). These kinds of interactive activities, supported by social media platforms, encourage the “everyday embedding” of reduction and cessation in the context of digital activity, providing tools that may promote self-reflection on smoking behaviors, increased self-awareness of the reasons for continued smoking, the benefits of quitting, and tips for successful cessation.

### Comparisons With Prior Work

To the best of our knowledge, there have not been previous studies of photo-based methods deployed as online tobacco interventions in the context of Facebook or other SNS. As such, there are several areas in which the current study findings provide unique contributions to the young adult tobacco cessation and eHealth intervention literatures. First, as participant-driven photography and other photo-based methods have seen increased prominence in health promotion research, a project like Picture Me Smokefree helps to make a case for innovation in the use of visual methods, in order to capitalize on widespread use of online platforms for uploading and sharing photos online, particularly among young people. While some youth-based tobacco control campaigns have incorporated user-created photos and video in innovative ways, this occurs primarily in the context of online contests and is typically not directly linked to measuring cessation outcomes or behavioral changes. While there may be high levels of user engagement and interactions that result from this digital content (eg, online voting), there remains a need for evaluation components that go beyond assessing short-term engagement. Likewise, as the results of participant-driven photography and other visual research have been descriptive, our project provides preliminary support for shifting participant-driven photography from a visual method that chronicles health problems, toward a scalable, sustainable intervention.

While we have emphasized that a unique aspect of this research project was that it included both active tobacco users and those who were in the process of, or had been successful at smoking cessation, the idea of bringing together these groups is not our invention. This strategy has been used by some of most successful youth anti-tobacco campaigns, including the US-based Truth initiative, which has from its inception been inclusive of youth that are actively smoking. For example, their online campaign materials have stated that “We are here to empower not to judge” and “This is about getting everyone on the path to ending smoking. It’s not about making fun of smokers. Or leaving them out. We love smokers. The more of them we can get on board, the closer we can get to ending teen smoking once and for all” [8]. Likewise, Canadian campaigns such as Quitters Unite have also positioned their online materials to be inclusive and welcoming to youth who may be actively smoking or resistant to quitting (eg, “Whether you smoke, have quit, or just want to support someone else to quit, there’s something here for you”) [56].

### Limitations

While these preliminary findings show good evidence for engagement, efficacy will need to be evaluated in a future controlled study to determine if this strategy leads to significant changes in smoking behavior and cessation outcomes pre- and post-intervention. The self-reported feedback from participants about changes in their smoking during and following their participation suggests that there is potential for this approach to motivate movement toward cessation for those who are still smoking and to reinforce being smokefree for young adults who had taken steps to quit tobacco.

An aim of this study was also to consider gender-related influences, and while we noted some apparent trends in how men and women participated in the intervention, further study is still needed. In the context of gender-specific groups, it is quite possible that we might observe different styles of interaction, support, and sharing between users if assigned to men-only, women-only, and mixed-gender photo groups. This is an area where the eHealth literature is nascent, albeit based on findings about how gender influences face-to-face support groups in health, prioritizing gender in digital and online health research is critical to determining how “gender matters” interconnect with people’s engagement and participation in the context of the virtual/online world.

### Conclusions

Picture Me Smokefree suggests that for young adults who smoke, an online context might provide the awareness of smoking behaviors that serves as the motivation for their beginning to contemplate quitting, even if the expectation of being completely and permanently smokefree may not yet be the priority for some people in this age group. Bringing together those who are actively smoking with those who are thinking about, or who have committed to quitting, to regularly share their experiences may be more appropriate for this age group as it more closely mirrors their social interactions in the “real-life” offline world, where those who smoke and those who do not frequently socialize. Likewise, in terms of gender, our participants expressed no preference for gender-specific groups or online communities, but rather stated that mixed-gender group mirrored their social interactions and relationships with other tobacco users who may be their partners, friends, and family.

## Multimedia Appendix 1

PowerPoint presentation on the Picture Me Smokefree intervention (2013).

## Abbreviations

RCT: randomized controlled trial
SNS: social networking site
WATI: Web-assisted tobacco interventions
