# Supplementary material for: Picture Me Smokefree: A Qualitative Study Using Social Media and Digital Photography to Engage Young Adults in Tobacco Reduction and Cessation
Source: J Med Internet Res. 2015 Jan 26;17(1):e27. doi: 10.2196/jmir.4061 (PMC4327185; doi:10.2196/jmir.4061)
Supplement: Supplementary file 1 [file jmir_v17i1e27_app1.pdf]

a place of mind
THE UNIVERSITY OF BRITISH COLUMBIA

Picture Me Smokefree

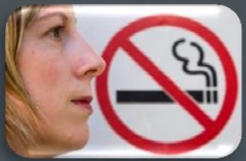

Using digital photography and social media to engage young adults

May 28, 2013

Rebecca Haines-Saah

Presentation to:
Clean Air Coalition of B.C - Knowledge Exchange Webinar
Vancouver, Canada

- How can we better engage youth and young adults?

- How can we engage with digital and visual media in new ways?

Guiding questions

SLIDES 3-5, 7-10 ON USER-GENERATED IMAGERY AND PHOTOGRAPHY ON SOCIAL MEDIA REMOVED DUE TO COPYRIGHT RESTRICTIONS

Slide 6

CAR SEATS for The Littles
Discussion, Education, and Recommendations

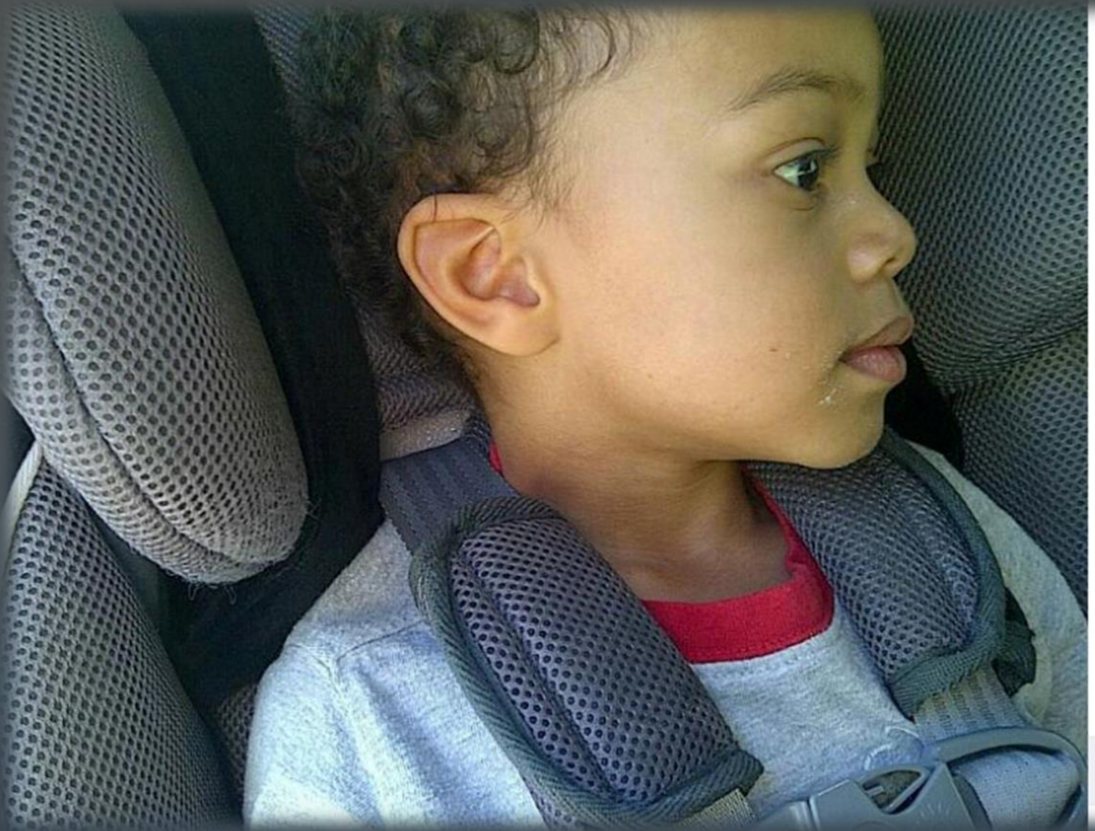

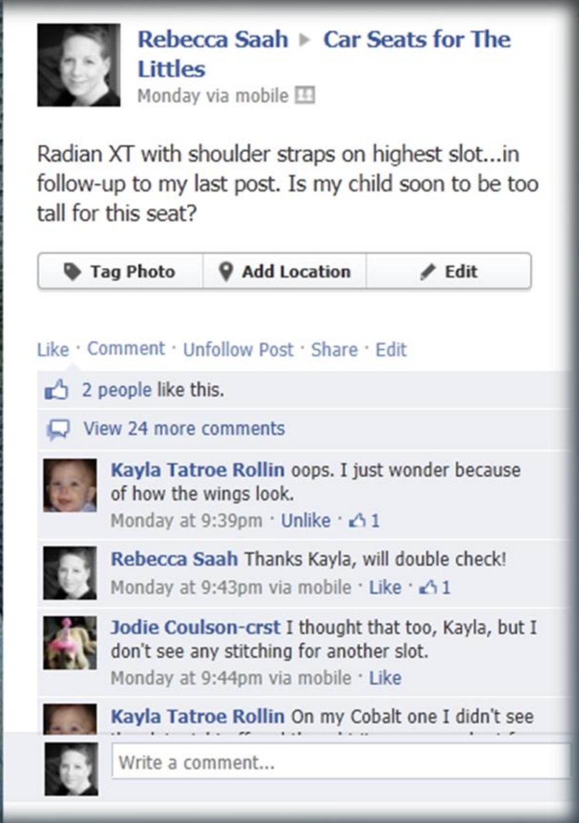

Facebook & health promotion

FACET

SMOKE, IN MY EYES

- Enables participatory methods
- Encourages participant reflection
- Empowers persons that smoke
- Engages with gender

Participant-driven photography

- Precedent for "WATI" in tobacco control
- Growth of photo methods in health research
- Promising pre-pilot study
- New funding for social media initiatives

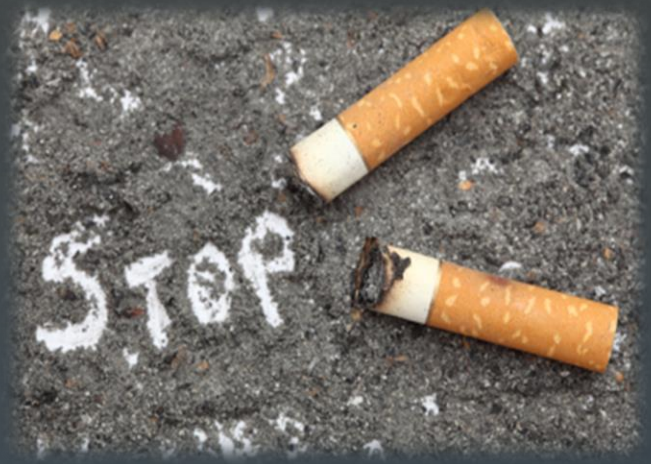

Rationale

Project Aims

- Support young adults in cessation
- Determine feasibility of a photo-based social media intervention
- Develop gender-sensitive programming
- Inform tobacco prevention campaigns for young adults

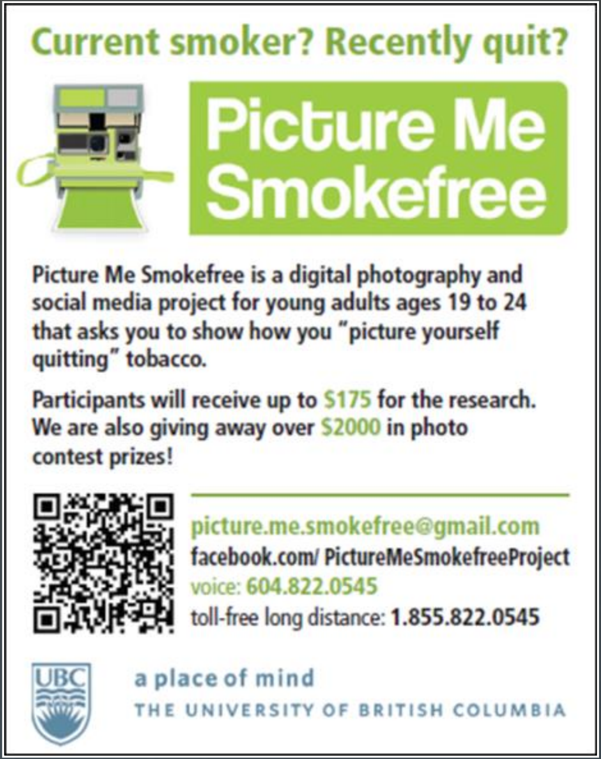

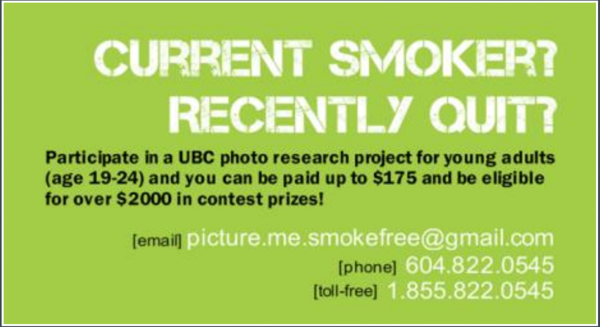

Picture Me Smokefree

Eligibility

- Age 19-24
- Live in British Columbia
- Literacy in English
- Have internet and camera access*

Project structure

- 1 brief sign-up visit (phone/in-person)
- Facebook group (1post per week x 12 wks)
- Brief online follow-up survey (39/60)
- *Optional*: one-to-one interview at completion (n=22; 10 men & 12 women)

Picture Me Smokefree

Recruitment

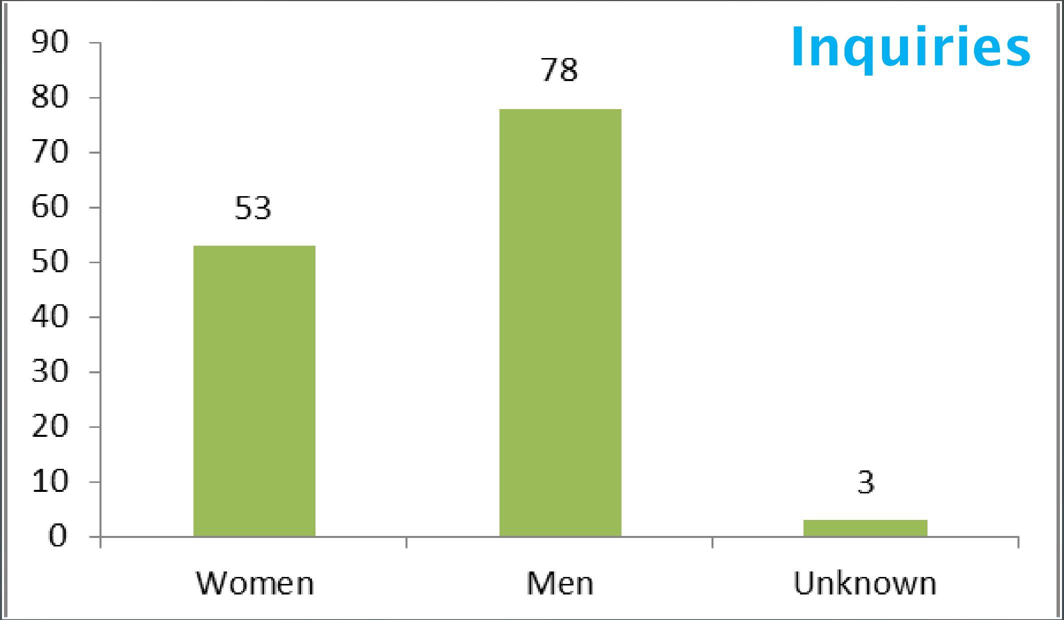

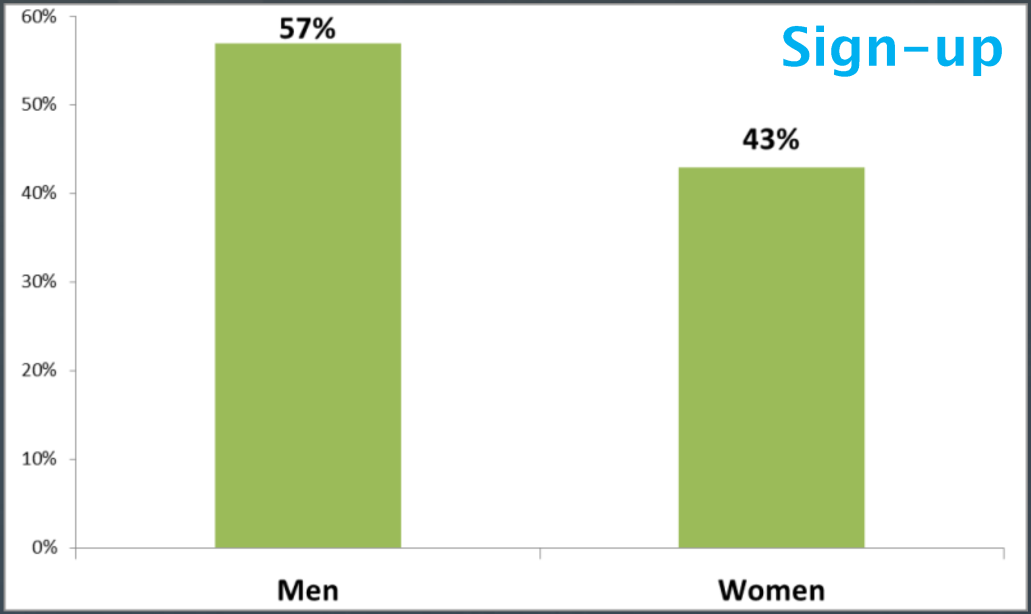

Smoking status @ sign-up

Slide 15

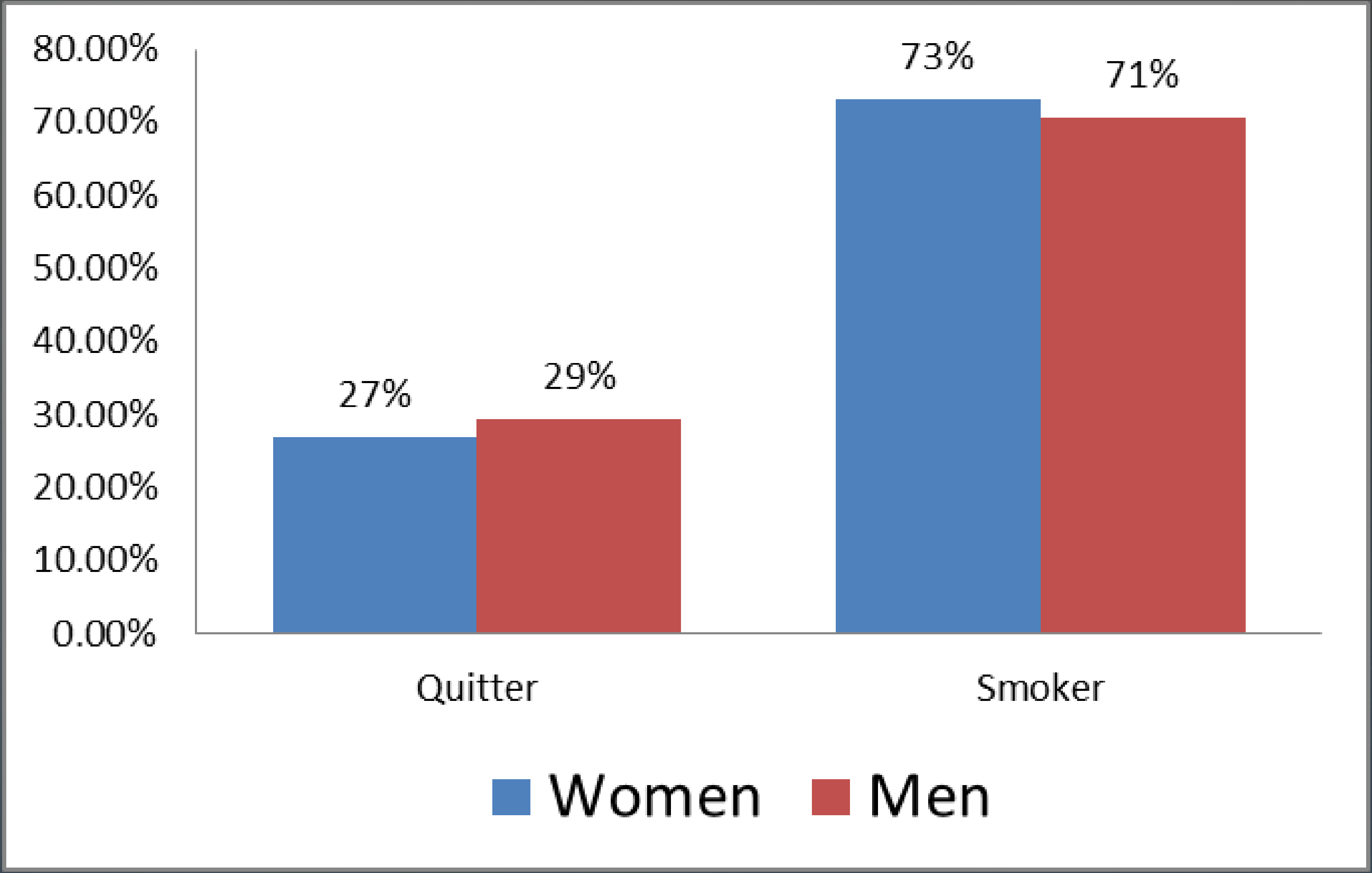

Participation

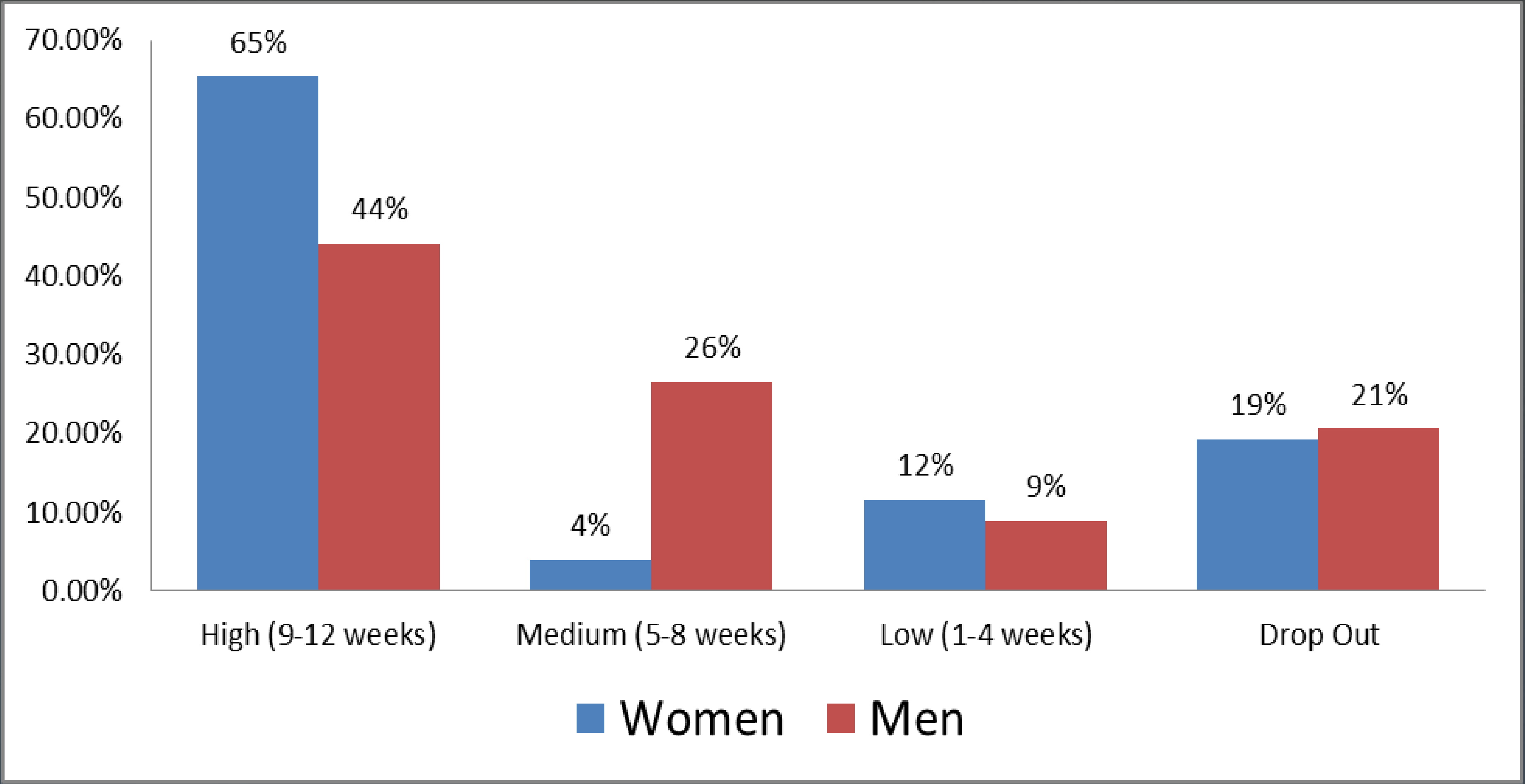

Comparison of number of weeks active by gender

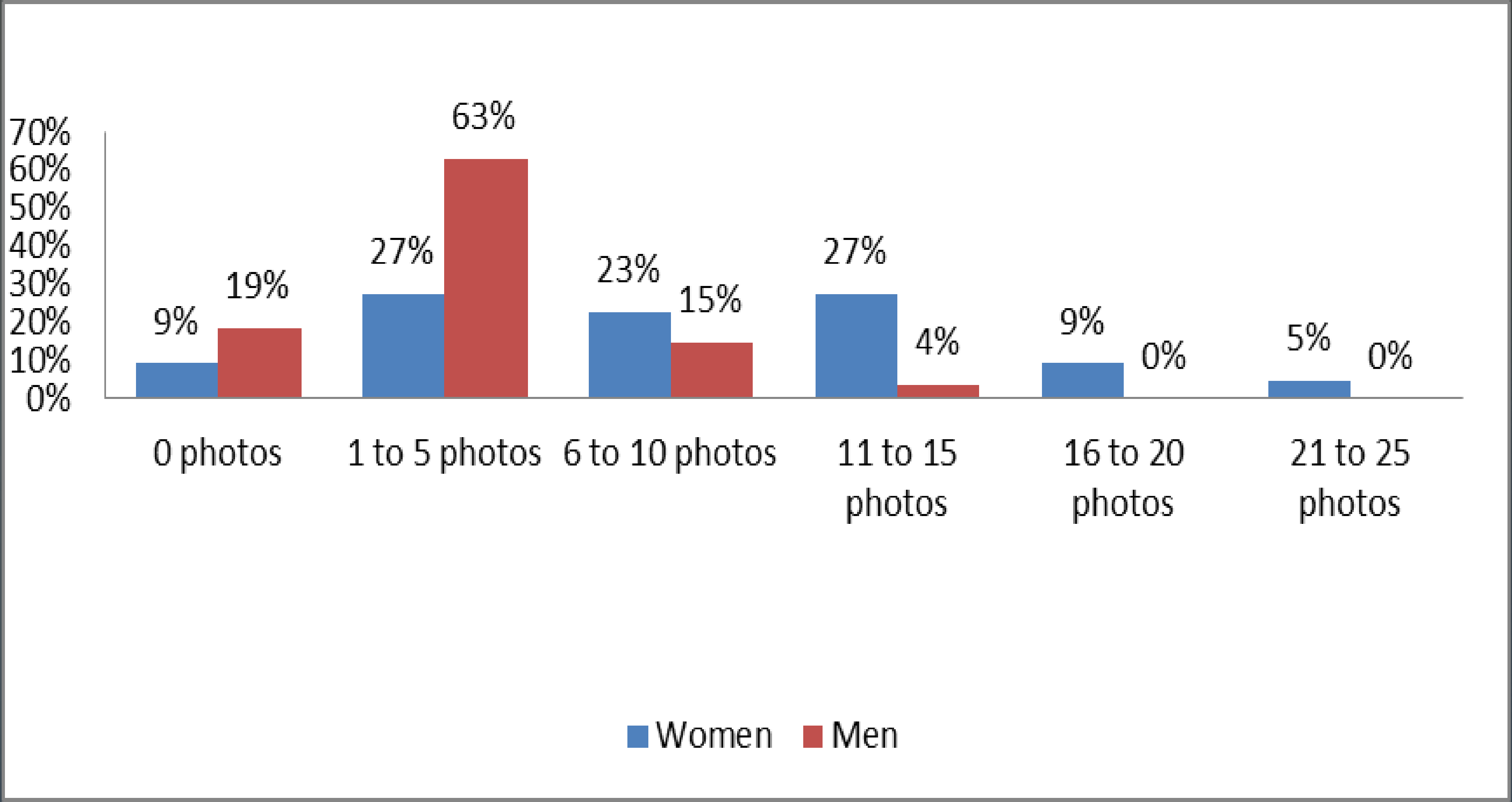

Comparison of number total photos posted by gender

Group posts

Indira CoRi
It is true! i don't really know if is a psycological thing but smoking used to make me feel more relaxed... I've been without smoking for 4 months and yesterday i was feeling stressed and ansious so i bought some :(I smoked one and then give away the rest with a smoker guy that i find in the street haha :(i'll keep on my smoke-free-actitud even if i'm on finals now ... Challenge Accepted! :)

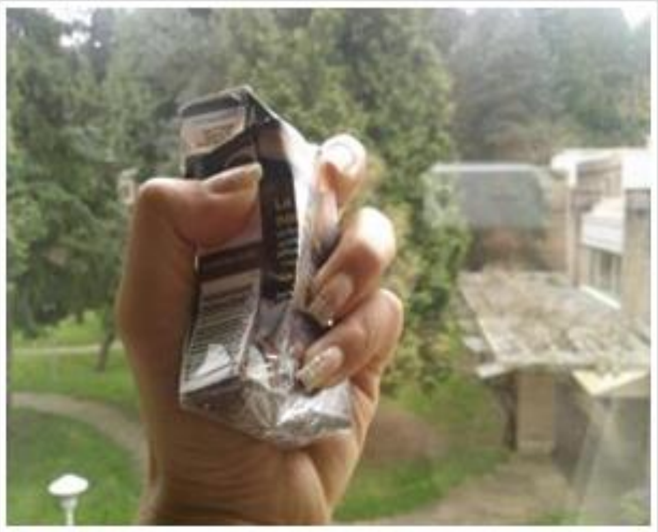

Like · Comment · Follow Post · Yesterday at 11:16am

[redacted] and 3 others like this.

[redacted]
Yesterday at 11:34am · Like · 1

[redacted] awesome self control :) good job
Yesterday at 3:53pm · Like · 1

Indira CoRi Thanks so much! it was so HARD i almost regret and walk back to ask if he could give me back the cigarettes lol
23 hours ago · Like

Group Themes
- Quit strategies
- Relapses
- Stress & triggers
- New pack labels
- Social/party context
- Family context
- *Some typically gendered imagery*

Group Tone
- Positive support
- Non-judgmental
- Harm reduction

Slide 19

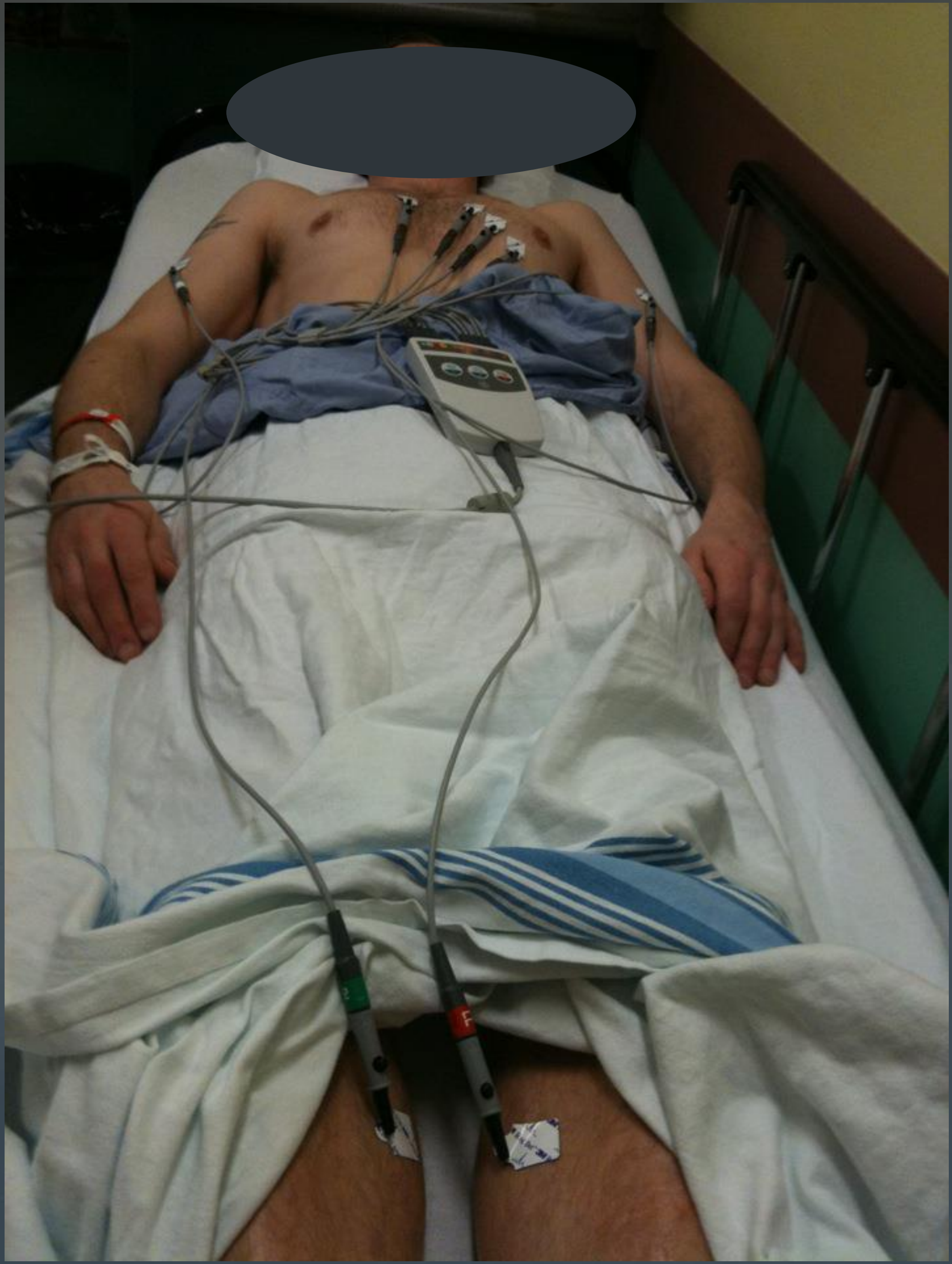

This is why I hate smoking. They said his heart is inflamed and smoking is contributing to it. I know that if we kept smoking this is where we will end up. I just keep asking why would I do this to myself?

When I was little, my parents smoked constantly…One day I was sitting in the backseat of the car (I was young) and my parents sat in the front. As they flung their ashes out the window, one landed in my Root beer. With my incredible knowledge of contamination and addictive properties of nicotine in cigarrettes (sic), I was convinced that I would now be addicted to root beer. I still am.

I thought I was naive at the time. When really, i was most naive 10 years later… When I thought smoking would relax me. Ignoring the picture right on the front of the pack "CIGARETTES HARM YOUR HEALTH", why is it the things that are supposed to make you feel good end up hurting you the most? (rootbeer included)

PARTICIPANT PICTURE OF ROOTBEER REMOVED DUE TO COPYRIGHT RESTRICTIONS

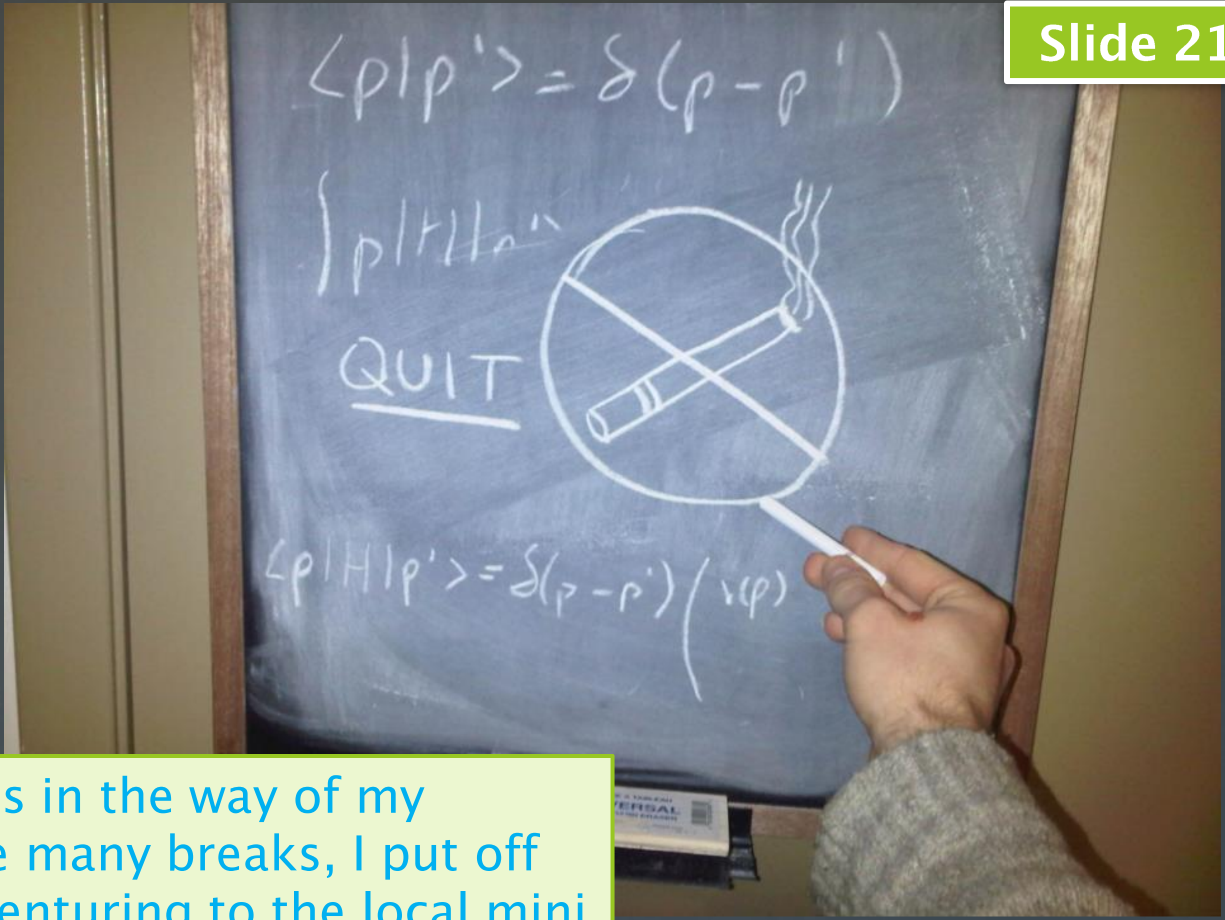

Sometimes smoking gets in the way of my academic pursuits. I take many breaks, I put off time for studying by adventuring to the local mini mart of gas station, I relax with the subtly deadly nature of tobacco. I feel that if I don't quit soon, it'll harm me in the long run, and my pursuit of becoming a professor will die off quit quickly (and literally). As soon as exams are out of the way, I will return to my non-smoking ways. But until then, I must cope..

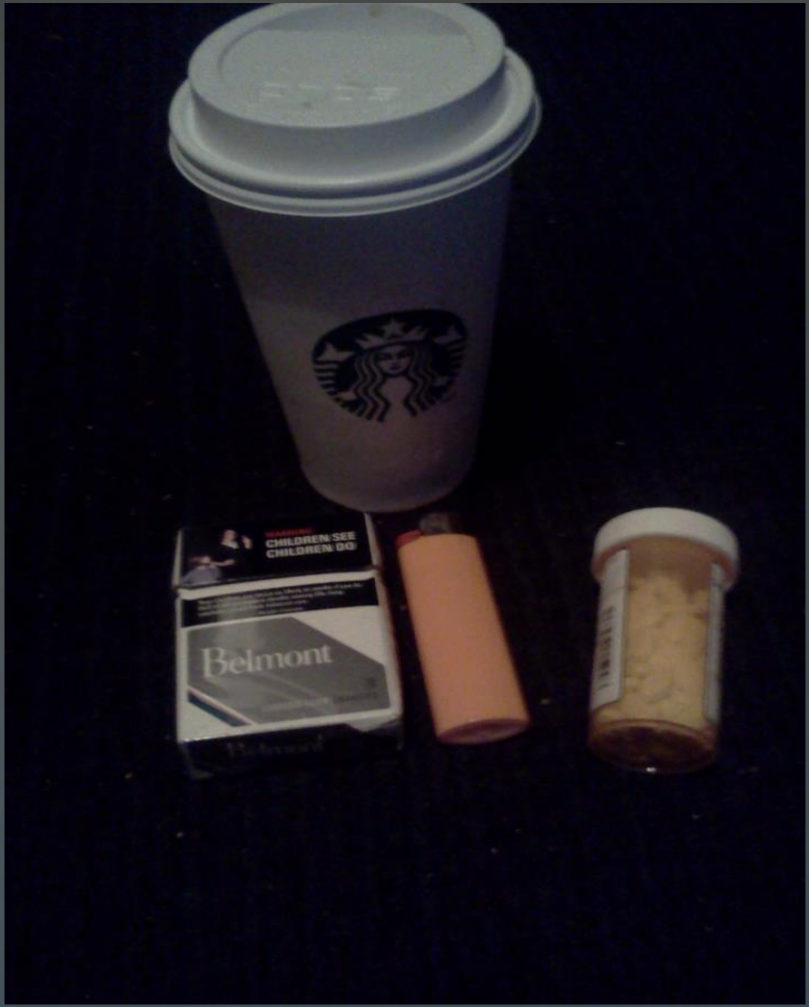

My everyday nessecities (sic), 2/5 are to do with cigarettes. I would like to eliminate those two things from my everyday .

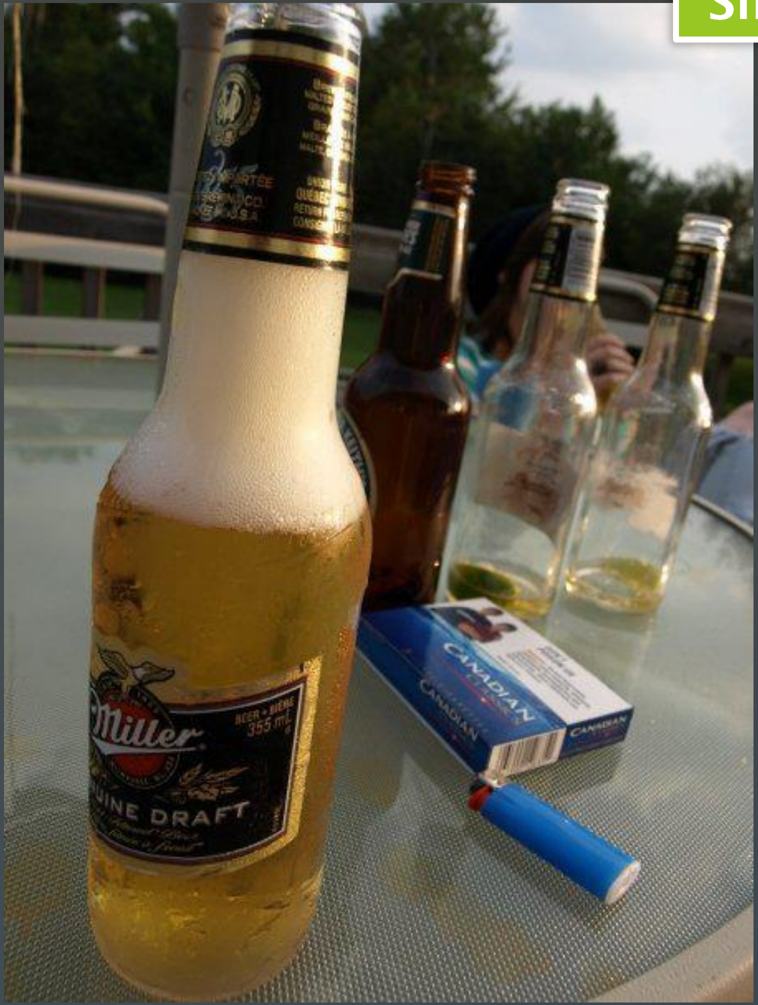

couple of beers n smoking: going to be the hardest habit to drop.

Participant feedback

FOLLOW-UP INTERVIEWS (n=22)

REFLCTION ABOUT SMOKING: I like the project because…it makes me aware of why I'm smoking, or like what makes me want to smoke and what like social situation and like just like anything that I'm doing that's like "oh I want a cigarette now."

PEER SUPPORT: I noticed like, the general atmosphere in the group, they seemed to be like fairly supportive and sometimes people, they will be like "oh I was doing so well and then I broke" and then everyone will be like "that's ok." (laughing)

REINFORCING CESSATION: Well, I find it helpful because, I quit smoking, it's like helpful to see that there are other people that are smoking but they are trying to quit. And they already quit or they are like struggling or in the middle.

GENDER: No strong preferences for a gender-segregated group; Enjoyed interaction with and learning from other genders

ONLINE ANONYMOUS FOLLOW-UP SURVEY (39/60)

On a scale of 1-10, please rate your overall experience as a participant in this project.

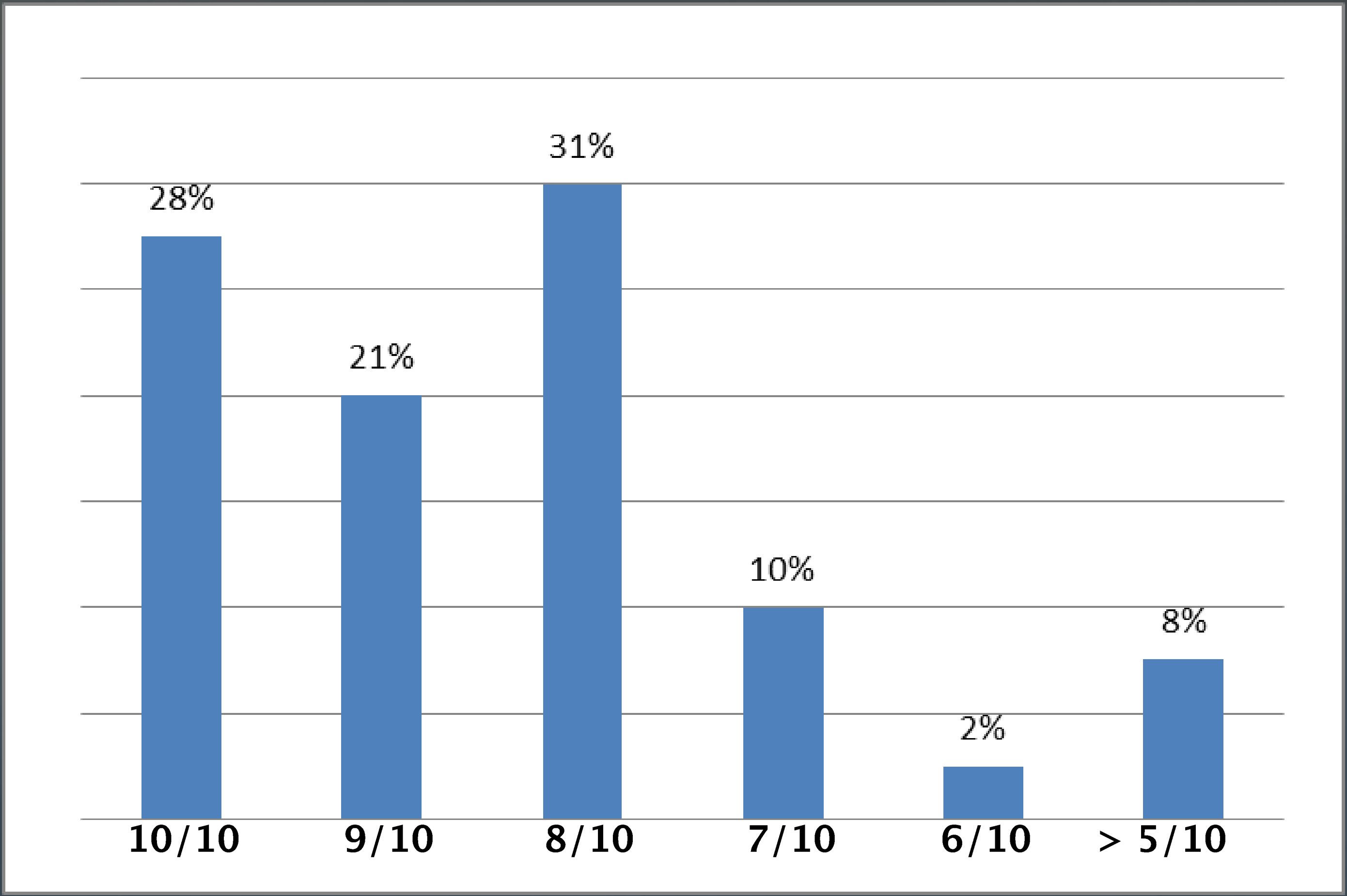

Would you participate again?

YES = 38
NO = 1

Did your smoking change while you were a participant in this project?

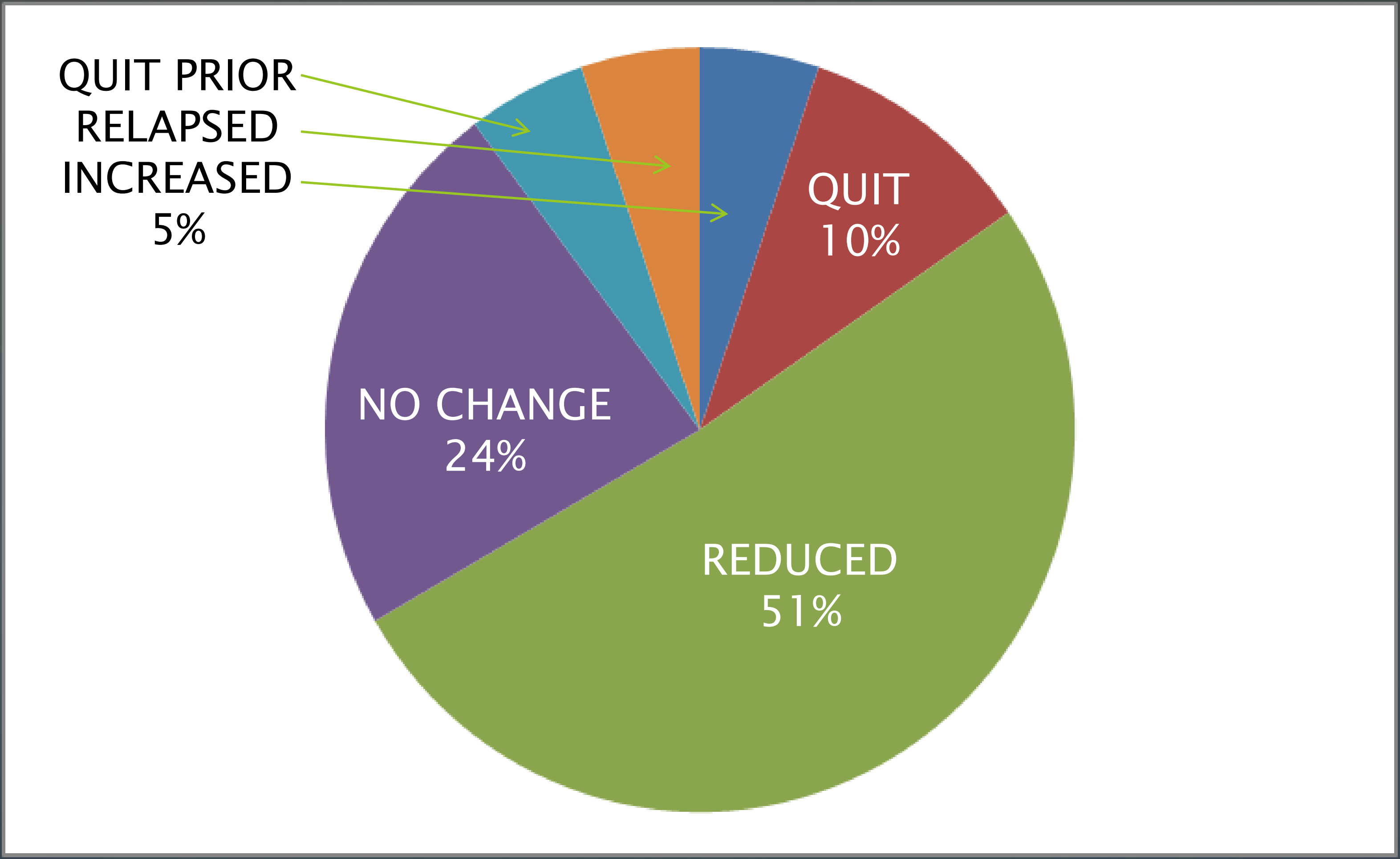

Like

- Support & connections
- New information
- Diversity of views
- Freedom/creativity
- Honesty/non-judgment
- Private group
- Easy: "only a mild hassle" "easy money"
- Reinforcement (daily reminder)

Dislike

- More interaction
- More interactive
- Too unstructured
- Need reminders to post
- Too busy/Lost interest
- Dislike Facebook
- Have offline meetings
- Allow non-smokers
- Difficulties creating images

Slide 27

…it's still been **a pleasure** to take part in this, as it acts as an open forum for smokers to discuss their addictions, and in that way it certainly helps. :)

Its nice to see I'm **not the only one** struggling with quitting smoking and all the things that make people think of smoking.

Thanks for having me in the group! It was a lot of fun and I'll probably stick around for a while longer. It's **really interesting** to see what others have to say/associate with smoking.

These last few weeks I have been really trying hard not to smoke, which is tough because of all the stress with school at the moment. Seeing (the study) poster really gave me an extra kick and **reminded me how important it is** to keep fighting my addictions.

I think **it helped in my efforts** to quit and I found it interesting to see what others had to say about smoking and quitting.

I have quit Facebook for the time being. **I have also quit smoking.**

Haven't smoked since my twelve weeks wrapped up. Instead, I've been eating way healthier, working out, and getting a ton more work done towards my career. Off to a great (re)start.

I am going to attempt what I believe to by my first serious attempt to quit smoking. I think **the seed of quitting was planted** in my mind this year when I took part in your project.

Feasibility issues

Recruitment

Remuneration

Sustainability

Access

Diversity

Content ownership

Cessation, reduction and engagement outcomes

- Potential for the visual as a point of engagement, reflection, & intervention
- Creating a supportive community of current *and* former smokers
- A “gender-sensitive” rather than “gender-specific” approach

Concluding thoughts

- Jack Boomer (Clean Air Coalition)
- Joan Bottorff (UBC)
- Katherine Frohlich (UdM)
- Rebecca Haines-Saah (UBC)
- John Oliffe (UBC)
- Kathryn Seely (CCSBCY)

Research team
